# Hybrid Light Harvesting Antenna Based on Si NWs and RuOs_2_ Dendrons for Near IR Light-Emission

**DOI:** 10.1021/acsomega.5c02574

**Published:** 2025-08-27

**Authors:** Giuliana Lazzaro, Maurilio Galletta, Ileana Ielo, Alessia Irrera, Maria Josè Lo Faro, Antonio Alessio Leonardi, Francesco Nastasi, Fausto Puntoriero

**Affiliations:** † Dipartimento di Scienze Chimiche, Biologiche, Farmaceutiche ed Ambientali, Università degli Studi di Messina and Centro Interuniversitario per la Conversione dell’Energia Solare (SOLARCHEM), via F. Stagno d’Alcontres 31, Messina 98166, Italy; ‡ CNR-IMM, viale F. Stagno d’Alcontres 31, Messina 98158, Italy; § Dipartimento di Fisica e Astronomia “E. Majorana”, Università di Catania, Via Santa Sofia 64, Catania 95123, Italy; ∥ CNR-IMM, Via Santa Sofia 64, Catania 95123, Italy

## Abstract

Silicon plays a crucial role in modern microelectronics and telecommunications. Recent advancements in nanotechnology have expanded its applications, particularly in photonics. Quantum confinement effects in silicon nanostructures, such as nanocrystals and nanowires (Si NWs), enable light emission in the visible-to-near-infrared (IR) spectrum at room temperature. Among these, Si NWs are particularly promising as they are compatible with existing microelectronic fabrication processes. Given the importance of near-IR light sources for telecommunications, research has focused on enhancing the silicon-based emission in this spectral range. This study presents the development of a hybrid light-harvesting antenna composed of quantum-confined Si NWs and Ru­(II)/Os­(II)-based dendrons. By leveraging energy transfer processes, these hybrid systems achieve near-IR emission at ∼920 nm with a remarkable 99.5% efficiency, as confirmed by lifetime measurements. The dye was anchored to the Si NWs via a carboxyl-functionalized bipyridine ligand, enhancing the stability of these hybrid systems. The demonstrated Si NWs/RuOs_2_ hybrid antenna offers significant advantages, including high energy transfer efficiency, stability, and compatibility with cost-effective silicon technology making these structures promising candidate for photonic applications.

## Graphical abstract

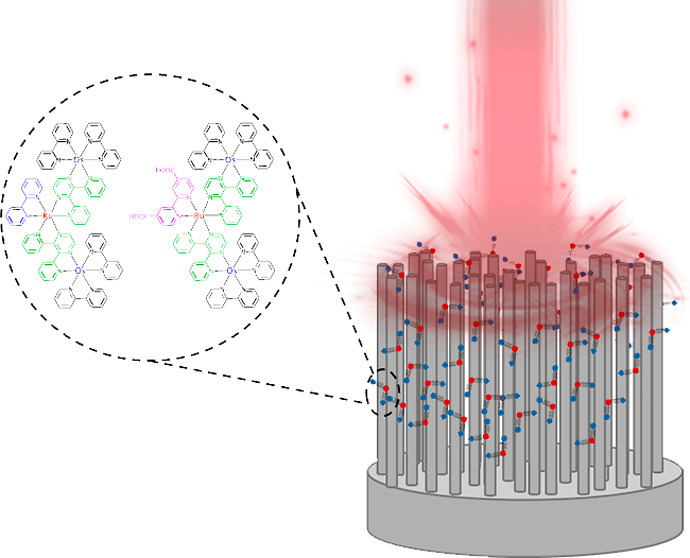

## Introduction

Silicon is one of the most important elements of nowadays technology, leading microelectronics and, considering its oxide, also telecommunication.^1^ During the last years, nanotechnology has opened novel opportunities for silicon in several fields such as microelectronics,^2–5^ energetics,^6–10^ sensing,^11–14^ and photonics.^15–18^ However, as far as photonics is concerned, silicon-based materials are mostly used as medium or substrate of photonic devices and a silicon-based light source that can be fully integrated in these systems is still a challenge.^19–24^ As is well-known, bulk silicon is an indirect band gap semiconductor that does not allow efficient emission of light at room temperature. However, nanotechnologies have shown that this limit can be surpassed by quantum confinement enabling a light emission at room temperature that can be engineered depending on the shape and size of the nanostructures.^25–29^ Quantum confined structures such as Si nanocrystals and Si nanowires (NWs) have already demonstrated interesting capabilities with light emission commonly peaked around 500–800 nm between visible and near-infrared region.^30–34^ Silicon nanocrystals are systems that have been extensively studied in literature but present several challenges, such as the difficulty of potential electrical pumping, their instability in air, and their integration into a flat fabrication architecture, like those typically used in microelectronics.^35–39^ A strategy to face these challenges has been based on systems based on SiO_2_ doped with Si nanocrystals. However, the adoption of a similar architecture implicitly limits their application in hybrid systems. On the other hand, Si NWs do not present similar issues and can be fabricated by approaches such as metal-assisted chemical etching (MACE) that are compatible with the current technologies of wafer fabrication adopted by microelectronics,^40–43^ making them suitable candidate of a silicon-based light source that can be fully integrated in a silicon photonic chip.

Telecommunication is mostly based on fused silica (SiO_2_) fibers, making the light attenuation characteristics of this material a critical point that should be taken into consideration in silicon photonics,^44,45^ making Si-compatible near IR light-emitting sources a critical resource in this field. Depending on the attenuation coefficient α (expressed in dB/km) measuring the loss of optical power as light propagates through the fiber.^46^ This attenuation strongly depends on the wavelength, and it is possible to identify 3 regions of main interest for photonics in the near IR region.^46^ The most known window is related to the long-range fiber optic communication associated with a wavelength around 1.55 μm where α ≈0.15 dB/km located close to the absolute minimum of the absorption losses of silicon, hence making it the standard one for long-range signal transport.^47^ The second window is around 1.3 μm in the presence of a relative minimum of the attenuation coefficient with α ≈0.3 dB/km and its mostly adopted for medium-range communication.^48,49^ The last window at about 900–1100 nm is positioned just below the silicon band gap (around 1.55 eV), and it is a compromise between an acceptable attenuation coefficient (for short distances) and the technological availability of low-cost detector and light sources in this range.^50^ This near-infrared window is commonly used in very short-range telecommunications or in optical devices as it allows a good light propagation for distances below tens of meters with the advantage of cost-effective devices.^51^ The window at about 900 nm is located at the tail of the Si NW light emission obtained by using a thin-film MACE approach.^52^

Numerous studies have been conducted in the past on hybrid systems obtained by coupling silicon nanocrystals with various chromophores.^53^ Specifically, energy transfer processes with efficiencies of up to 65% were reported for such systems, utilizing chromophores at millimolar concentrations.^53^ The design of hybrid systems involves the engineering of organic molecules (e.g., BODIPY dyes^54^) on Si NWs that exhibit excited states which can be easily modulated and can function as energy acceptors with respect to the nanostructured material. The same strategy has been proven successfully with the synthesis of a hybrid system consisting of silicon nanowires onto which a Ru­(II) and Os­(II) tetranuclear metal complex was physisorbed, reaching an energy transfer efficiency from the nanowires to the tetranuclear complex of up to 93%.^52^ The interest in Si NWs lies on the availability of fabrication methods that are compatible with the microelectronics industry and on top of commercial wafers.^55,56^ Moreover, while silicon nanocrystal is a single emitter, silicon nanowires are characterized by a high number of radiative recombinations along their length, thus enabling a very efficient long-range energy transfer process. The interest in Ru­(II) and Os­(II) polypyridine complexes is due to their very interesting photophysical, photochemical, and redox behavior.^57^ As well-known, when these complexes are part of a single supramolecular structure, new properties appear such as photoinduced energy transfer^57^ with interesting perspectives for water splitting,^58,59^ artificial photosynthesis,^60^ solar cells,^61^ and so on.^62,63^ In fact, based on the choice of the bridging ligands between the metal subunits and the system’s topology, understood as the organization in terms of space, time, and energy between the various blocks that make up the supramolecular system, it is possible to model structures that exhibit the desired photophysical properties (light absorption, luminescence, redox potentials, etc^64^). A hybrid antenna able to combine both the advantages of quantum confined Si nanowires and Ru­(II)/Os­(II) polypiridine complexes can be of great interest to tackle the challenge of a silicon-based light source in the near-infrared.

In this article, the manufacturing of a high energy transfer efficient hybrid antenna of quantum confined Si NWs and Ru/Os-based trinuclear dendrons (RuOs_2_) has been demonstrated. The aim of the current work is to demonstrate how the emission can be further shifted into the near-infrared (NIR) region, which is particularly relevant for photonics and short-range telecommunication applications. This was achieved through the engineering of a trinuclear RuOs_2_-based dye, which, when integrated into a hybrid silicon nanowire structure, exhibits emission at around 920 nm. A mixed trinuclear complex of Ru­(II) and Os­(II), previously investigated,^65^ was selected for this study on novel hybrid structures based on Si NWs. An analogous system was synthesized in which the bipyridine ligands directly coordinated to the Ru­(II) center and was functionalized with two carboxylate groups. This modification was introduced to evaluate the differences in efficiency and stability between the chemisorbed hybrid and the physisorbed system. As expected from the dendrons engineering, a light emission at about 920 nm is obtained in these systems while the Si NW peak emission at 670 nm is strongly suppressed in the hybrid structures. In this light-harvesting antenna, Si nanowires act as energy donors to the RuOs_2_ metal dendrons (energy acceptors), demonstrating a remarkable efficient energy transfer that reach 99.5% according to the lifetime measurements.

Additionally, we explored a carboxylic-modified RuOs_2_-based dendron (dcRuOs_2_) to enhance the stability of the hybrid structure and significantly reduce the fabrication time. In our previous work,^52^ adsorption times of 41 and 22 h were required for Ru_4_ and Ru_3_Os, respectively. In contrast, the physisorption time for RuOs_2_ was reduced to 24 h and further shortened to just 8 h with dcRuOs_2_ chemisorbed dye. The use of the carboxyl functional group for the chemisorption protocol guaranteed a higher stability of the hybrid antenna that retained its light emission after several washing procedures. These Si NWs/RuOs_2_ hybrid systems provide key benefits such as compatibility with cost-effective silicon technology, high stability, efficient energy transfer, the remarkable ability to operate with low molecular concentrations, and light-emission in the near IR considered strategic for photonic applications.

## Experimental Section

### Materials

4″ Si commercial wafers (4 in.) were purchased from University Wafer. HF, H_2_O_2_, and gold etching were acquired from Merk. Ethanol, water, CH_3_CH, CH_2_Cl_2_, MeOH, diethyl ether, NH_4_PF_6_, AgNO_3_, aluminum oxide, and 2,2′-bipyridine were purchased from Sigma-Aldrich and used without further purification.

### Fabrication

Synthesis of the metal complexes [(bpy)­Ru­{(μ-2,3-bis-pyridil-pyrazine)­Os­(bpy)_2_}_2_]­[PF_6_]_6_ (RuOs_2_) and [Cl_2_Ru­{(μ-2,3-bis-pyridil-pyrazine)­Os­(bpy)_2_}_2_]­[PF_6_]_6_ (Cl_2_RuOs_2_) were performed as previously reported in the literature by the authors.^65^ The 4,4′-dicarboxy-2,2′-bipyridine was reprepared accordingly to literature.^66^

Synthesis of [4,4′-dicarboxy-2,2′-bipyridineRu­{(μ-2,3-bis-pyridil-pyrazine)­Os­(bpy)_2_}_2_]­[PF_6_]_6_ (dcRuOs_2_) was performed as follows. A solution of [Cl_2_Ru­{(μ-2,3-bis-pyridil-pyrazine)­Os­(bpy)_2_}_2_]­[PF_6_]_4_ (30.3 mg, 0.0136 mmol), 4,4′-dicarboxy-2,2′-bipyridine (6,6 mg, 0.027 mmol), and AgNO_3_ (5 mg, 0.0347 mmol) was refluxed for 48 h under an inert atmosphere, in a 1:1 (v/v) mixture of ethanol and water (5 mL). The reaction was conducted in the dark. Upon cooling, the mixture was centrifuged to remove the precipitate of AgCl. Excess solid NH_4_PF_6_ was then added, resulting in the formation of a violet precipitate, which was isolated by filtration. The obtained solid was further purified by column chromatography on neutral aluminum oxide (2.5 cm diameter, 20 cm length, aluminum oxide activity 1), using a 9:1 (v/v) CH_2_Cl_2_/MeOH elution. The last violet band collected was concentrated via rotary evaporation. The residue was dissolved in a minimal volume of acetonitrile (CH_3_CN), and the product was reprecipitated by the addition of diethyl ether. The overall yield was 60%. Elemental analysis: calcd (found) for C_80_H_60_F_36_N_18_O_4_Os_2_P_6_Ru (%): C, 35.74 (36.02); H, 2.25 (2.26); N, 9.38 (9.32).

Si NWs were fabricated by a thin-film MACE by using a 2 nm gold layer as a catalyst. A proper cleaning by a two-step sonication in (i) isopropanol and (ii) water was initially performed, finally drying the wafer by a nitrogen flux. After the first cleaning, a 2 min UV–ozone treatment followed by an etching in a 2.5 M HF aqueous solution was then performed to ensure the removal of possible biological contaminants and silicon native oxide, respectively. The 2 nm Au discontinuous film was deposited by electron beam evaporation on the previously treated commercial silicon wafer. After the evaporation process, the sample was immersed in a 5 M/0.025 M HF/H_2_O_2_ aqueous solution for about 20 min. After the etching of the silicon underneath the metal layer, 2.5 μm long Si NWs were obtained and the sample was finally treated with a gold etching solution for 1 min to remove the Au catalyst. The Si NWs/RuOs_2_ and Si NWs/dcRuOs_2_ hybrid systems were obtained by immersing the nanowires at room temperature in a solution of acetonitrile containing RuOs_2_ and dcRuOs_2_, at a concentration of 4.30 × 10^–5^ M and 4.32 × 10^–5^ M, respectively.

### Characterization

Scanning electron microscopy (SEM) images were acquired by using a ZEIS SUPRA 25 with an InLens detector operating with an electron beam at 5 kV.

UV/vis absorption spectra of RuOs_2_ and dcRuOs_2_ in acetonitrile were recorded with a Jasco V-570 spectrophotometer. The instrumental uncertainty of the measurements is ±2 nm.

Emission spectra of RuOs_2_ and dcRuOs_2_ in acetonitrile were recorded with a Horiba-Jobin-Yvon FluoroMax-P spectrofluorometer coupled with a Hamamatsu R3896 photomultiplier. The instrumental uncertainty of the measurements is ±5 nm.

Photoluminescence spectra of Si NWs and Si NWs/RuOs_2_ hybrid structures were acquired by a Horiba Jobin Yvon HR800 microRaman spectrometer equipped with a −70 °C Peltier cooled CCD operating in a backscattering configuration with a 100X (NA = 0.9) objective and exciting the system with a 488 nm Ar^+^ laser line. The power onto the sample plane was measured to be around 100 μW. The photoluminescence emission of the hybrid structure was deconvolved into 2 Gaussian contributions of Si NWs and RuOs_2_, respectively. The integrated signals refer to the integration of the Gaussian area of the respective contribution. The photoluminescence lifetime measurements of Si NWs were acquired by using an Edinburgh OB-900 time-correlated single-photon-counting (TCSPC) spectrometer, employing a Hamamatsu PLP2 laser diode as pulse (wavelength output, 408 nm; pulse width, 59 ps).

## Results and Discussion

The complex [(bpy)­Ru­{(μ-2,3-dpp)­Os­(bpy)_2_}_2_]­[PF_6_]_6_ (RuOs_2_) was synthesized according to our previous studies^65^ (more details in the Experimental Section), whereas the new [(4,4′-dicarboxy)­bpyRu­{(μ-2,3-dpp)­Os­(bpy)_2_}_2_]­[PF_6_]_6_ (dcRuOs_2_) was prepared to the goal of this work, see Figure 1a. In this nomenclature, bpy and 2,3-dpp represent 2,2′-bipyridine and 2,3-bis­(2-pyridyl)­pyrazine, respectively.

**Figure 1:**
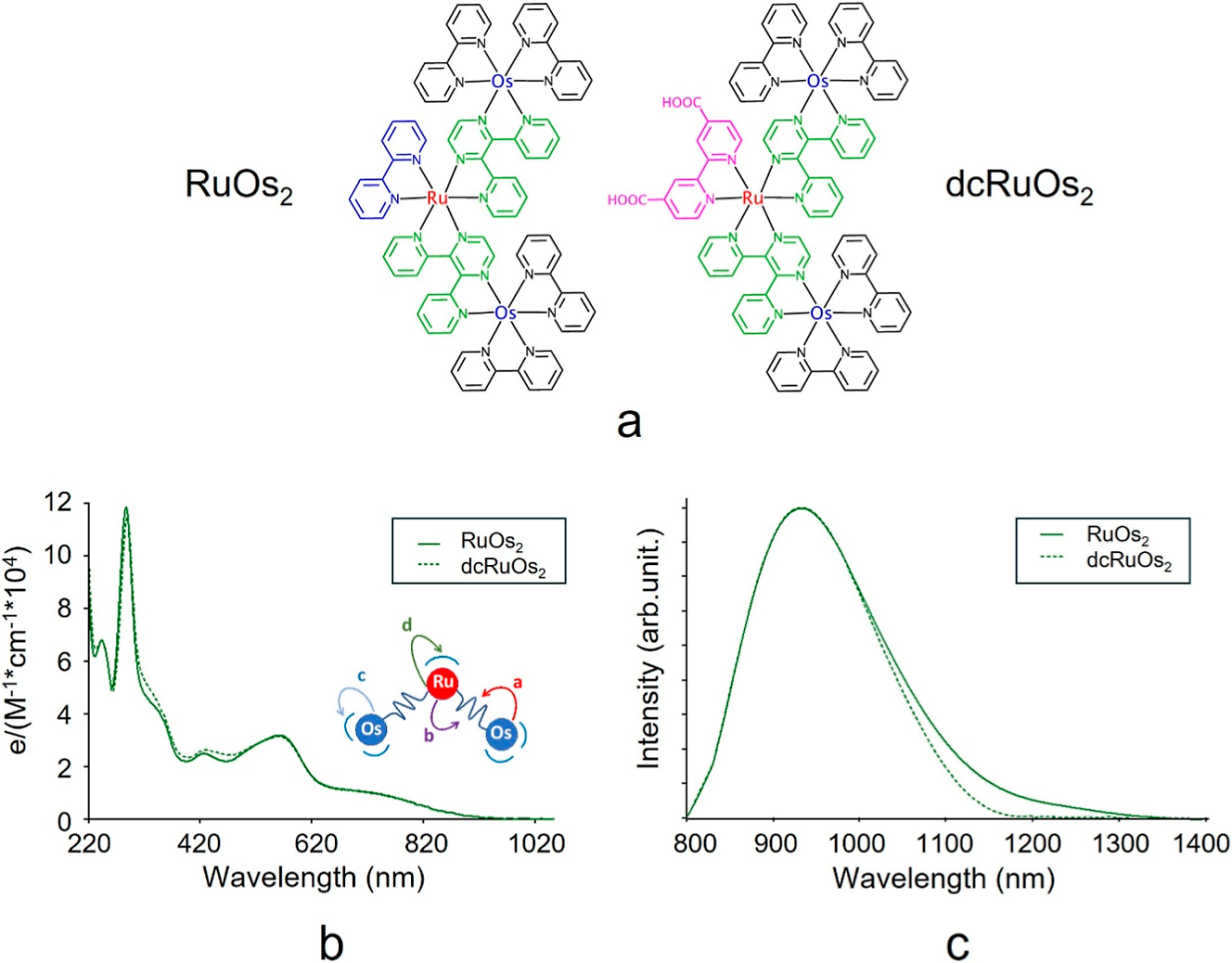
(a) Chemical structures of RuOs_2_ and dcRuOs_2_, charge and counterions are omitted for clarity. (b) Absorption spectra of the two RuOs_2_-based complexes in acetonitrile solution. In the inset, the schematic of the RuOs_2_ and dcRuOs_2_ transitions is shown. (c) Phosphorescence of the two dendrons in acetonitrile solution.

The absorption and emission spectra of RuOs_2_ and dcRuOs_2_ in acetonitrile solution, as shown in Figure 1b,c, respectively, are nearly identical, suggesting that the carboxylate groups present in dcRuOs_2_ do not significantly impact the optical properties of the system. More specifically, regarding the absorption spectra, a slightly different profile can be noticed in the region between 300 and 500 nm, presumably due to the presence of carboxylic groups on the bipyridine coordinating Ru­(II) metal ion. The bands in the UV region are attributable to the ligand-centered transitions of the coordinated ancillary and bridging ligands.^65^ The visible region is essentially dominated by a series of bands due to spin allowed metal to ligand charge transfer (MLCT) transitions, whose decreasing energy order d > c > b > a is illustrated in the inset of Figure 1b.^65^ The absorption bands observed at wavelengths longer than 730 nm can be attributed to spin-forbidden metal-to-ligand charge transfer (MLCT) transitions, which exhibit enhanced intensity due to the strong spin–orbit coupling arising from the heavier Os­(II) metal center. Consistent with the absorption spectra, the emission profiles are also almost completely superimposable. In both trinuclear complexes, regardless of the excitation wavelength, emission originates from the low-lying ^3^MLCT state involving the Os­(II) metal center and the bridging 2,3-dpp ligand.^65^ This demonstrates that the carboxyl groups on the bipyridine ligand coordinating the central Ru­(II) subunit have no influence on the lowest-energy state responsible for the emission. Both trinuclear complexes (bpyRuOs_2_ and dcbpyRuOs_2_) are found to be stable in acetonitrile.^65^

Preparation of Si NWs was performed using the thin-film MACE technique, an approach that involves chemical etching catalyzed by a gold thin layer at room temperature, characterized by an easy chemical metal removal following the nanowires’ fractal array formation (more details are given in the Experimental Section). In Figure 2a, a schematical representation of the fabricated Si NWs is reported while the prepared 2.5 μm long Si NWs are shown in a cross section scanning electron microscope (SEM) image in Figure 2b.

**Figure 2:**
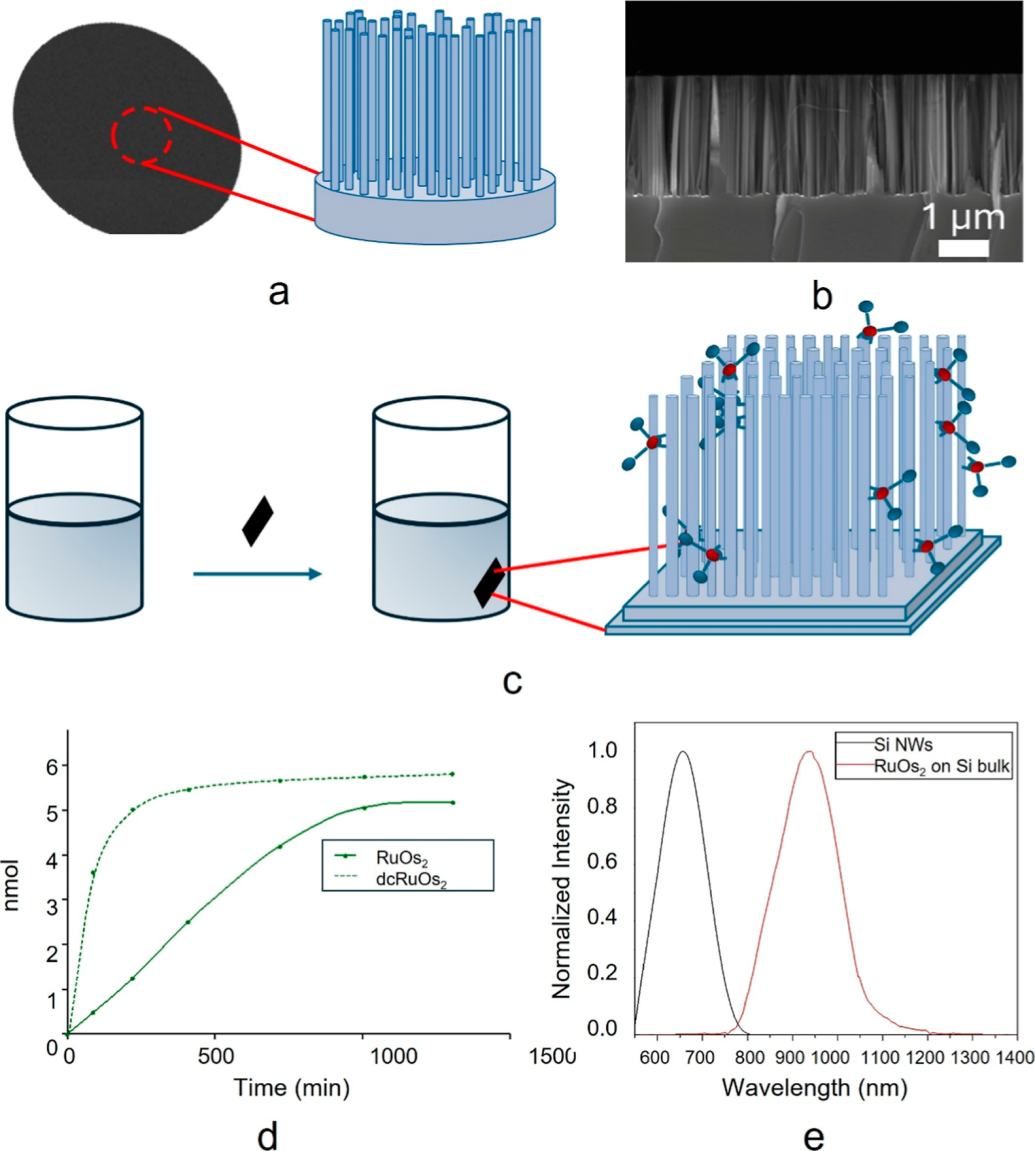
(a) Schematic representation of the fabricated Si NWs. (b) Cross-section SEM image of as-grown Si NWs. (c) Schematic representation of the Si NWs/RuOs_2_ hybrid structure synthesis. (d) Number of moles deposited as a function of time for both physisorption (RuOs_2_) and chemisorption (dcRuOs_2_) processes. (e) Normalized luminescence of the Si NWs and RuOs_2_ deposited in a Si bulk substrate as a reference for the emission in the solid phase.

After the Si NW fabrication, Si NWs/RuOs_2_ and Si NWs/dcRuOs_2_ hybrid systems were prepared by immersion as described in more details in the Experimental Section and schematized in Figure 2c. In particular, Si NW samples were immersed in a 4.30 × 10^–5^ M acetonitrile solution of RuOs_2_ or dcRuOs_2_ acquiring the absorption spectra of the liquid phase to attest when the deposition process was saturating (Figure 2d). Indeed, the processes of physisorption and chemisorption were monitored spectroscopically. In the bpyRuOs_2_ solution, we have a physisorption process in which the molecules of the dye simply adhere to the surface of Si NWs due to electrostatics forces.^52^ In the case of dcRuOs_2_ solution, it forms an ester bond between carboxylic groups of the bpy and the native silicon oxide on the nanowires surface. Absorption spectra were recorded for dye solutions at regular time intervals until no further changes in the absorption spectra were observed. This allowed for the determination of kinetic profiles for both the physisorption and chemisorption processes, and the amounts of metal complex removed from the solution were calculated. In the first case, the amount was found to be 5.1 nmol, while in the second case, it was 5.95 nmol (Figure 2d). As the deposited amount is pretty similar for both dyes, a 3-fold reduced time leading to 8 h of process has been attested for the chemisorption of dcRuOs_2_, demonstrating the interest of a similar strategy.

The increase in the number of moles of complex removed from the solution over time indicates the process of transferring the dye from the solution to the nanowires. The amount of substance transferred from the solution to the substrate depends on the migration of the complexes within the Si NW system, and it becomes constant once a thermodynamic equilibrium is reached between the solution and the nanowires. It is important to note that thermodynamic equilibrium was reached in one-third of the time for dcRuOs_2_, indicating that the equilibrium is strongly favored toward infiltration. This is likely due to the carboxyl groups, which facilitate anchoring to the nanostructures, making the system less prone to release due to strong interactions between the dye and the Si NWs. Furthermore, by considering the amounts of complex absorbed in relation to the exposed surface area of the nanowires, we estimate the dye density per unit area to be in the range of few picomol/cm^2^ for both hybrid systems (more information is given in the Supporting Information).

The samples, after infiltration, were dried under a gentle nitrogen flux. Figure 2e shows the emission spectra of bare Si NWs and RuOs_2_ adsorbed on Si bulk used as a reference for the dendron emission in the solid state where no change in the emission peak is attested.

The Si NWs/RuOs_2_ and Si NWs/dcRuOs_2_ hybrid systems obtained after 18 and 8 h, respectively, were then further analyzed acquiring the photoluminescence at room temperature in the visible and infrared by exciting the system at 488 nm (more details are given in the Experimental Section).

The luminescence spectra of the two Si NWs/RuOs_2_ and Si NWs/dcRuOs_2_ hybrid systems are shown in Figure 3a,b, respectively. The Si NW emission peak is located at around 660 nm, while the trinuclear complex emission is visible at about 940 nm. It is worth noting that the Si NW emission is shown multiplied by a factor of 1 × 10^7^ as its signal is very low after the dendron deposition. The integrated peak of the dendron at 920 nm is shown in Figure 3c for both Si NWs/RuOs_2_ and Si NWs/dcRuOs_2_. In the case of Si NWs/dcRuOs_2_, a slight 12% increase is attested. To show the stability of the chemisorbed hybrid structure, both Si NWs/RuOs_2_ and Si NWs/dcRuOs_2_ were washed in acetonitrile several times. A negligible change was attested by the light-emission of the dendrons for Si NWs/dcRuOs_2_ (Figure 3c), demonstrating the stability of the structure. On the other hand, a negligible signal of RuOs_2_ was measured after washing the physisorbed structure Si NWs/RuOs_2_. This further demonstrates the interest in the chemisorbed dcRuOs_2_ structure compared to the physisorbed RuOs_2_ one.

**Figure 3:**
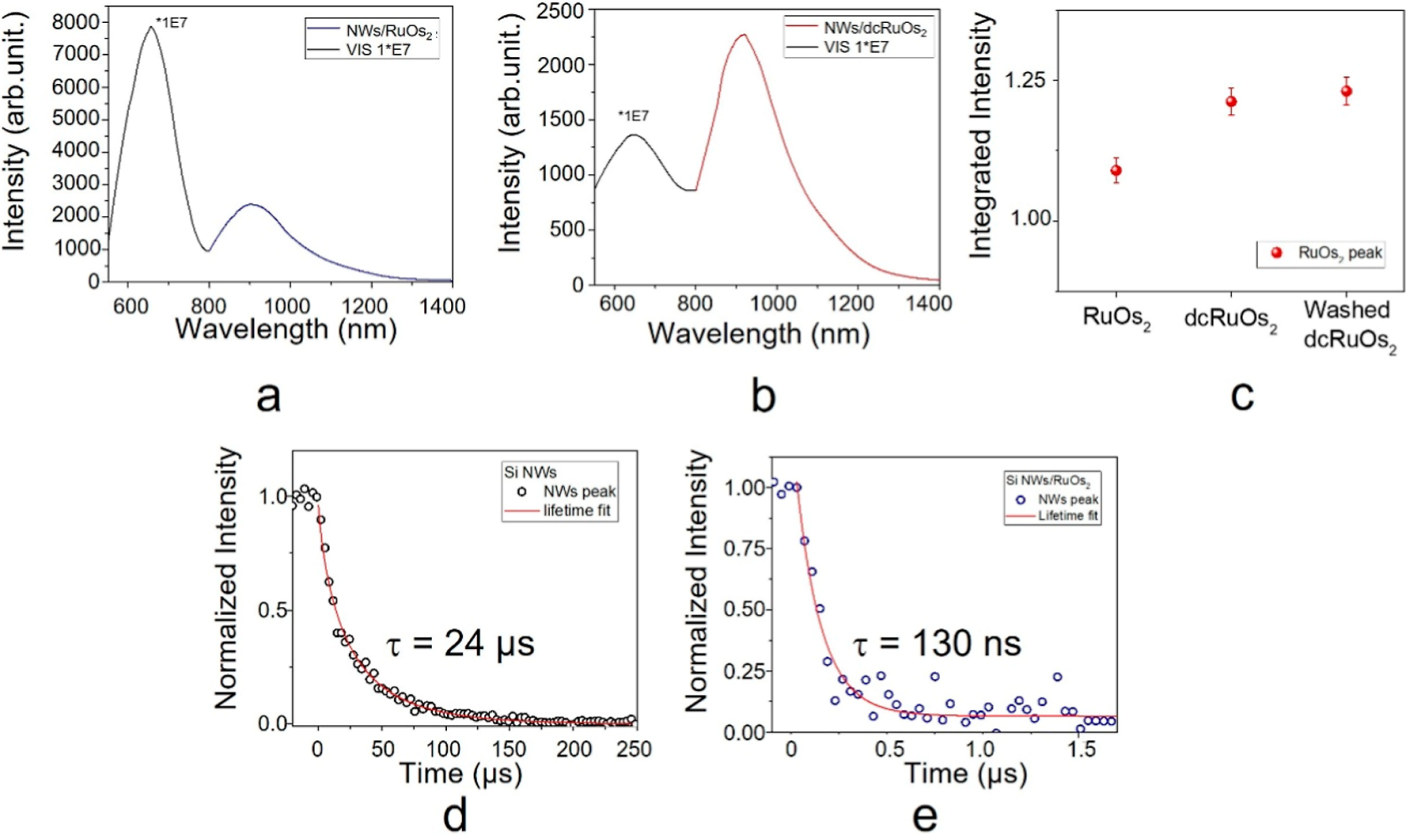
Photoluminescence spectra of (a) Si NWs/RuOs_2_ and (b) Si NWs/dcRuOs_2_. (c) Integrated dendrons peak emission for Si NWs/RuOs_2_ and Si NWs/dcRuOs_2_ before and after the washing tests. Luminescence lifetime measurement for the Si NW emission peak at 660 nm for bare Si NWs and Si NWs/dcRuOs_2_ in (d,e), respectively.

As can be seen from the spectra in Figure 3a,b, there is a near-total quenching of the nanowire luminescence in favor of the low-energy-emitting state of the trinuclear complexes. Considering the small amount of complex chemisorbed (a few picomol/cm^2^) and the absorption of the dye at the excitation wavelength (488 nm), the luminescence obtained is only from a negligible part due to the direct absorption of the metal complexes, so we can hypothesize that the energy absorbed by the nanowires is efficiently transferred to the metal complexes.

The presence of energy transfer was investigated by performing lifetime measurements of the Si NW peak emission at 660 nm for the as-grown NWs (Figure 3d) and the hybrid Si NWs/dcRuOs_2_ structure (Figure 3e). By fitting the obtained lifetime curve, it was found that the nanowires reduced their lifetime from 24 μs (bare nanowires) to 130 ns (hybrid system).

By considering the lifetimes of the Si NW peak with and without dyes, is it possible to estimate both the rate constant (*K*_en_) and the efficiency of the energy transfer process (η) with the following equations^52^

1 $$K_{en}=\frac{\tau_{{BNW}_{s}}-\tau_{{HNW}_{s}}}{\tau_{{BNW}_{s}}\cdot \tau_{{HNW}_{s}}}$$

2 $$\eta =1-\frac{\tau_{{HNW}_{s}}}{\tau_{{BNW}_{s}}}$$

For both hybrid systems, *K*_en_ is 9.9 × 10^6^ s^–1^ (about 100 ns), and η is around 99.5%; this represents the highest efficiency ever reported for systems of this type, even 6.5% higher than the previous system reported in the literature with tetranuclear Ru_4_ or RuOs_3_ dendrimers.

The overall optical results are summarized in Table 1, showing the absorption, luminescence, and lifetime data for both the precursors and the two hybrid systems.

**Table 1:** Absorption and Photophysical Data

compound	absorption λ_max_/nm	ε/M^–1^ cm^–1^	luminescence λ_max_/nm	τ/ns
RuOs_2_ ^a^	425	22,200	926	
	559	31,900		
	714	9300		
dcRuOs_2_ ^a^	425	21,800	926	
	559	31,900		
	714	9300		
Si NWs	276		670	24,000
	420			
Si NWs/RuOs_2_			670	130
			920	
Si NWs/dcRuOs_2_			660	130
			920	

a Data recorded in MeCN solution.

On the basis of these results, the new hybrid system showing an efficient energy interaction between silicon nanowire and RuOs_2_ is demonstrated with near-infrared emission that can be of interest for photonics applications.

The reported hybrid antenna based on Si NWs and trinuclear RuOs_2_ complexes are compared in Table 2 with the literature of hybrid systems involving silicon nanostructures and organic light-emitting molecules. It is worth noting that our works are the only ones reporting silicon nanostructures as donors, and a remarkable energy transfer efficiency up to 99.5% with a 6.5% efficiency enhancement has been attested in this work.

**Table 2:** Comparison of Hybrid Antenna Based on Si Nanostructures

material	acceptor	donor	ET efficiency (%)	ref
Si NCs/anthracene	Si NCs	Anthracene	70	^67^
Si NCs/pyrene	Si NCs	pyrene	≈90	^68^
Si NWs/metal dendrimers	Ru_4_ Ru_3_Os	Si NWs	93	^69^
Si NWs/metal dendrimers	dcRuOs_2_	Si NWs	99.5	this paper

## Conclusion

In this study, we synthesized and characterized two trinuclear Ru­(II)–Os­(II) complexes, RuOs_2_ and dcRuOs_2_, and successfully integrated them into the Si NW hybrid systems. Spectroscopic analysis revealed that the presence of carboxyl groups in dcRuOs_2_ did not significantly affect the optical properties in solution but played a crucial role in surface anchoring. The chemisorption process enabled by these functional groups led to a more stable hybrid system compared with the physisorbed RuOs_2_ counterpart. Kinetic analysis demonstrated that the chemisorption process reached equilibrium in just 8 h—three times faster than the physisorption process. Additionally, washing tests confirmed that dcRuOs_2_ remained firmly attached to the Si NWs, while RuOs_2_ was easily removed, further proving the enhanced stability conferred by covalent bonding.

Photoluminescence studies revealed near-total quenching of the Si NW emission with energy efficiently transferred to the metal complexes. Lifetime measurements confirmed a drastic reduction from 24 μs (bare NWs) to 130 ns (hybrid system), indicating a highly efficient energy transfer. Calculations yielded an energy transfer efficiency (η) of 99.5%, the highest ever reported for this type of system, surpassing previous literature values by 6.5%.

These results highlight the potential of RuOs_2_ and dcRuOs_2_ as effective energy acceptors in Si NW-based hybrid systems. The enhanced stability and superior energy transfer efficiency of dcRuOs_2_ make it particularly promising for applications in optoelectronics and photovoltaic devices. Future studies will explore further functionalization strategies to optimize the performance and expand the applicability of these hybrid materials.

## Supplementary Material



## Notes

Comparative properties of Si NWs/trinuclear or tetranuclear hybrid systems, optical properties of Si NWs and RuOs_2_ trinuclear complexes, surface coverage of Si NW hybrid antennae, and estimating Förster Radius (PDF)
