# Supplementary material for: Hybrid Light Harvesting Antenna Based on Si NWs and RuOs2 Dendrons for Near IR Light-Emission
Source: ACS Omega. 2025 Aug 27;10(35):39606–14. doi: 10.1021/acsomega.5c02574 (PMC12423881; doi:10.1021/acsomega.5c02574)
Supplement: Supplementary file 1 [file ao5c02574_si_001.pdf]

Supporting Information

Hybrid Light harvesting antenna based on Si NWs and $RuOs_2$ dendrons for near IR light-emission

Giuliana Lazzaro,[a] Maurilio Galletta,[b,] Ileana Ielo,[a] Alessia Irrera,[b] Maria Josè Lo Faro,[c,d] Antonio Alessio Leonardi,[a,*] Francesco Nastasi,[a] Fausto Puntoriero[a]*

a) Dipartimento di Scienze Chimiche, Biologiche, Farmaceutiche ed Ambientali, Università degli Studi di Messina and Centro Interuniversitario per la Conversione dell'Energia Solare (SOLARCHEM), via F. Stagno d'Alcontres 31, Messina, 98166 Italy
b) CNR-IMM, viale F. Stagno d'Alcontres 31, Messina 98158, Italy
c) Dipartimento di Fisica e Astronomia "E. Majorana", Università di Catania, Via Santa Sofia 64, Catania 95123, Italy
d) CNR-IMM, Via Santa Sofia 64, Catania 95123, Italy
* Corresponding authors M.G. maurilio.galletta@cnr.it, A. A. L. anleonardi@unime.it.

Comparative properties of Si NWs/trinuclear or tetranuclear hybrid systems

In Ref. [1], tetranuclear dendrimers were employed to develop a hybrid light source, exhibiting dye emissions centered around 740 nm for the Ru_4-based compound and 800 nm for the Ru_3Os-based structure. These systems represented the first antenna configuration in which Si nanowires (Si NWs) acted as energy donors and the dye molecules as acceptors, achieving an efficient energy transfer of approximately 90%.

The aim of the present work is to demonstrate how this emission can be further shifted into the near-infrared (NIR) region, which is particularly relevant for photonics and short-range telecommunication applications. This advancement was made possible through the engineering of a trinuclear $RuOs_2$-based dye, which, in the current hybrid Si NW structure, exhibits an emission peak around 920 nm.

Additionally, we investigated a carboxylic acid–modified $RuOs_2$-based dendron ($dcRuOs_2$) to enhance the stability of the hybrid structure and significantly reduce fabrication time. In our previous work, adsorption times of 41 hours and 22 hours were required for Ru_4 and Ru_3Os, respectively. In contrast, the physisorption time for $RuOs_2$ in this study was reduced to 24 hours, and further decreased to just 8 hours for chemisorbed $dcRuOs_2$.

Table 1 compares the obtained Ru(II)- and Os(II)-based organometallic dendron hybrid systems with those reported in Ref. [1], highlighting advancements in both energy transfer efficiency and deposition time. Notably, the systems incorporating trinuclear complexes exhibit enhanced energy transfer efficiency, reaching up to 99.5%, compared to a maximum of 93% achieved by the tetranuclear Ru_3Os complex. Furthermore, the use of $RuOs_2$ complexes enables a shift in the emission

wavelength, pushing further into the near-infrared (NIR) region, an area of particular interest for short-range telecommunications and photonics applications. It should be highlighted that the chemisorption strategy not only improves the stability of the hybrid system but also significantly reduces the deposition time by at least a factor of three compared to the previously studied complexes.

Table S1

	**Ru_3Os**	**Ru_4**	**$RuOs_2$**	**$dcRuOs_2$**
**Energy Transfer efficiency (%)**	93	90-93	99.5	99.5
**λ_{em} (nm)**	800	745	926	926
**Anchoring strategy**	Physisorption	Physisorption	Physisorption	Chemisorption
**Deposition time**	22h	41h	24h	8h
**REF**	[1]	[1]	This work	This work

Optical properties of Si NWs and $RuOs_2$ trinuclear complexes

$RuOs_2$ and $dcRuOs_2$ absorption slightly differs as shown in Figure 1b in the main text. For this reason, the only $dcRuOs_2$ absorption spectrum, normalized to the 540 peak, has been compared with the normalized Si NW photoluminescence in Figure S1 showing a small overlap between the two spectra in a region of minimum absorbance for the trinuclear dendron complex.

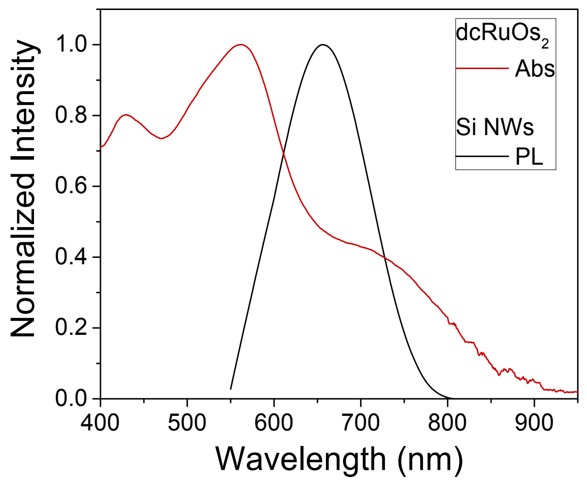

Figure S1. Spectral overlap between $dcRuOs_2$ normalized (peak at 560nm) absorption spectrum and normalized Si NW photoluminescence.

Surface coverage of Si NW hybrid antennae

The surface coverage was calculated by relating the adsorbed amount to the estimated surface area of the Si NW array, determined based on the average nanowire diameter and their density[1]. The adsorbed mass was derived from the change in absorption for the 560 nm peak of the solution before and after deposition. Specifically, the amount of adsorbed material was found to be 5.1 nanomoles for $RuOs_2$ physisorption (after 24h) and 5.95 nanomoles for $dcRuOs_2$ chemisorption (after 8h). The surface area was estimated assuming a filling factor of about 60% (FF 0.6) has previously reported for these Si NW array [2] and an average diameter of 7 nm [3,4] and a length of 2.5 μm, with each Si nanowire modeled as a cylindrical structure. The filling factor represents the occupied fraction of the surface in the substrate plane by the Si NWs. Its area may be considered as the base surface of the Si NWs times the NW number.

The exposed surface area can be obtained considering the Si NW occupied area on the substrate plane according to the filling factor times the ratio between this area (the base area of a cylinder) and the high aspect ratio of these cylindrical structures is negligible. Indeed, the length of the average Si NWs l=2.5μm is about 3 order of magnitude higher than the average radius r=3.5nm meaning that the average top circle area is negligible compared to the average surface lateral area of the cylinder use to represent a Si NW (eq. 1)

$$l \gg r \rightarrow 2\pi r l \gg \pi r^2 \qquad (1)$$

The ratio between the base area of a Si NW and its lateral one is

$$\frac{2\pi r l}{\pi r^2} = \frac{2l}{r} \approx 1430 \qquad (2)$$

A 2cm^2 with a 0.6 FF corresponds to a 1.2 cm^2 of occupied area that times the area ratio of Si NW calculated in eq 2 will give:

$$S = 1.2cm^2 * 1430 \approx 1716\, cm^2 \qquad (3)$$

Considering about 5.5 nanomoles (an average between 5.1 and 5.9 nanomoles) of dendrons we got a surface coverage of

$$S_{cov} = \frac{moles}{surface} = \frac{5.5\, nanomoles}{1716 cm^2} \approx 3\, picomoles/cm^2 \qquad (4)$$

Estimating Förster Radius

In order to gain insight into the non-radiative energy transfer processes occurring in the system composed of silicon nanowires (Si NWs) functionalized with photoactive complexes, we carried out a quantitative analysis of the energy transfer efficiency (E) based on the Förster resonance energy transfer (FRET) theory. The analysis was informed by the experimentally observed reduction in

photoluminescence (PL) lifetimes of the donor species upon functionalization, which is indicative of energy transfer from the donor (Si NWs) to the acceptor moieties (metal complex units).

According to Förster theory, the energy transfer efficiency ($E_{Efficiency}$) can be expressed by the following equation:

$$E_{Efficiency} = \frac{{R_0}^6}{{R_0}^6 + {R_{DA}}^6} \quad (5)$$

where R_0 represents the Förster radius - defined as the distance at which the energy transfer efficiency is 50% — and R_{DA} denotes the donor–acceptor separation distance.

Using the experimental energy transfer efficiency value we obtained a Förster radius of:

$$R_0 = 14.6\,\text{Å} \quad (6)$$

To estimate R_{DA}, we considered the spatial organization of the molecular components based on molecular modeling and known structural parameters of the functionalized system. Specifically, the distance between the donor (Si NW surface) and the acceptor (the lowest-energy metal-to-ligand charge transfer, ^{3}MLCT, center involving the osmium complex and the 2,3-dpp bridging ligand) was approximated to be 6.3 Å. This value reflects the presumed proximity between the photoluminescent center of the Si NWs and the acceptor transition dipole, localized near the osmium center and the bridging ligand.

The spectral overlap integral J was calculated by numerically integrating the normalized donor emission spectrum with the molar extinction coefficient (ε) of the acceptor over the relevant wavelength range. The resulting value of the overlap integral was determined to be:

$$J = 4.145478 \times 10^{-13}\, cm^6 \cdot mmol^{-1} \quad (7)$$

This result implies that the donor–acceptor separation (6.3 Å) falls well within the effective range for FRET to occur, supporting the hypothesis that the observed luminescence quenching is attributable to efficient non-radiative energy transfer between the Si NWs and the osmium-based acceptor units.

References

(1) Leonardi, A. A.; Nastasi, F.; Morganti, D.; Lo Faro, M. J.; Picca, R. A.; Cioffi, N.; Franzò, G.; Serroni, S.; Priolo, F.; Puntoriero, F.; Campagna, S.; Irrera, A. New Hybrid Light Harvesting Antenna Based on Silicon Nanowires and Metal Dendrimers. *Adv Opt Mater* **2020**, *8* (24). https://doi.org/10.1002/adom.202001070.

(2) Lo Faro, M. J.; Ruello, G.; Leonardi, A. A.; Morganti, D.; Irrera, A.; Priolo, F.; Gigan, S.; Volpe, G.; Fazio, B. Visualization of Directional Beaming of Weakly Localized Raman from a Random Network of Silicon Nanowires. *Advanced Science* **2021**, 2100139. https://doi.org/10.1002/advs.202100139.

(3) Leonardi, A. A.; Faro, M. J. Lo; Miritello, M.; Musumeci, P.; Priolo, F.; Fazio, B.; Irrera, A. Cost-Effective Fabrication of Fractal Silicon Nanowire Arrays. *Nanomaterials 2021, Vol. 11, Page 1972* **2021**, *11* (8), 1972. https://doi.org/10.3390/NANO11081972.

(4) Leonardi, A. A.; Arrigo, A.; Lo Faro, M. J.; Nastasi, F.; Irrera, A. 2D Fractal Arrays of Ultrathin Silicon Nanowires as Cost-Effective and High-Performance Substrate for Supercapacitors. *Advanced Energy and Sustainability Research* **2024**, *5* (9), 2400080. https://doi.org/10.1002/AESR.202400080.
